# Safety climate and readiness for implementation of evidence and person centered practice – A national study of registered nurses in general surgical care at Swedish university hospitals

**DOI:** 10.1186/s12912-016-0174-2

**Published:** 2016-09-13

**Authors:** Camilla Olsson, Anna Forsberg, Kristofer Bjerså

**Affiliations:** 1 Department of surgery Östra sjukhuset, Sahlgrenska University Hospital, Gothenburg, Sweden; 2 Department of Health Sciences, Medical Faculty at Lund University, Lund, Sweden; 3 Department of Transplantation and Cardiology at Skåne, University Hospital, Skåne, Sweden; 4 Division of Nursing Science Faculty of Medicine and Health Sciences, Linköping University, Linköping, Sweden; 5 Department of Surgery, Clinical Sciences, Sahlgrenska Academy, University of Gothenburg, Gothenburg, Sweden

**Keywords:** Patient safety, Safety climate, Person centered care, Surgical nursing, Surgical care

## Abstract

**Background:**

The rationale behind this study is the increasing research on relationships between patient safety, evidence based practice and person centered care, and the growing interest in outcomes of surgical patients. The aim of this study was to explore the safety climate and readiness to implement evidence-based and person centered care as perceived by registered nurses in Swedish surgical care.

**Methods:**

The design was an exploratory, cross-sectional survey carried out in a national Swedish context. Data were collected through the Safety Attitudes Questionnaire (SAQ – Short form) and the Context Assessment Index (CAI).

**Results:**

In total, 1570 questionnaires were distributed, of which 727 were returned, giving a response rate of 46.3 %. The results revealed that in general, the safety climate in Swedish surgical care is positively related to readiness for evidence-based and person centered care, although specific management and cultural factors may be more sensitive and represent targets for improvement.

**Conclusion:**

This study presents new knowledge regarding the safety climate and readiness to implement evidence based practice and person centered care in general surgical wards in university hospitals and indicates important associations between these two areas. While RNs generally reported positive job satisfaction and a good team work culture in their units, there were indications that improvements in organizational management are needed.

**Electronic supplementary material:**

The online version of this article (doi:10.1186/s12912-016-0174-2) contains supplementary material, which is available to authorized users.

## Background

There is increasing and significant evidence of the relation between how surgical care is organized and performed throughout the perioperative process and the impact on mortality and morbidity [1–3].

A recurring subject in the health care literature is the importance of promoting safe work practices by viewing the safety climate as a crucial institutional priority. There is a need to develop work environments where safety has high priority. An important first step is to describe the safety climate in acute care hospitals [4–7]. Research [5, 8, 9] indicates that the safety climate should be regarded as a constellation of profession-specific sub-climates that are best measured at the nursing unit level. Previous studies conducted at this level mainly cover intensive care units, operating theaters or emergency departments [10, 11]. A consequence is that we lack knowledge about the safety climate in surgical or medical units where a substantial number of patients are treated during their hospital stay. Nurses is the largest health care profession globally, and its members mainly working at the bedside, close to the patient, and around the clock, with a holistic perspective on the patient and her/his care. In Sweden, where this study was performed, 107, 000 registered nurses (RNs) were employed in the health care sector in 2013 [12], making nurses the dominant profession in the health care system. In this study we therefore focused on nurses because previous research indicates that frontline caregivers are in the best position to provide information about the safety climate in nursing units [6, 10, 11].

In the United States of America, the Institute of Medicine [13] stated that nurses play a critical role in providing safe care and identified health care management practices required for generating a positive patient safety culture. The practices mentioned were creating a culture of openness with regard to the reporting and prevention of errors, as well as including the staffs in decision making concerning work design and work flow. Sexton et al. [14] drew parallels between aviation and medical safety and emphasized organizational cultural attitudes toward team work behavior and openness about error for endorsing a constructive safety climate. They compared physicians and nurses with aviation professionals. The latter reported less team openness about error, less team collaboration, and additional acceptance for working when fatigued. Furthermore, Aiken et al. [15, 16] linked lower nursing staff levels to increased patient mortality and a significant relationship was established between staff nurses’ empowerment, supportive nursing practice environments and perceptions of a positive safety climate [17]. When exploring the quality and strength of the patient safety climate in medical-surgical units, Hughes et al. [5] found that obligation to safety among the nurses was the most strongly positive characteristic of the safety climate in the units investigated. Balancing duties with safety compliance was the only area in which the climate quality was poor. Finally, Armstrong, Laschinger and Wong [18] demonstrated a significant relation between the quality and nature of hospital nurses’ work environments and the level of the patient safety climate in those same environments. In summary, there is support in the literature for the connection amongst empowering work environments that promote professional practice and the presence of a positive patient safety climate.

An interesting organizational example is an investigation of the outcomes of >180,000 surgical cases by the National Surgical Quality Improvement Program (NSQIP) in the US. Findings revealed a significant increase in 30-day mortality for those undergoing elective, in-patient surgery on a Friday as opposed to those operated on between Monday and Wednesday [19]. However, the difference was only evident in patients admitted to general wards, while those admitted to the intensive care unit or were day-surgery patients were not affected. Zare et al. [19] suggest that reduced staffing levels in general wards on weekends might have an adverse effect on patient outcomes. As revealed by further work from the NSQIP, a significant difference in surgical mortality was identified between institutions, despite a similar case mix adjusted morbidity rate [20]. Suggestions about how to interpret this have been offered, such as “failure to rescue” in hospitals with a higher mortality rate might be associated with context related issues, e.g., medical and nursing staffing levels, which was also suggested by Aiken et al. [15, 16]. In addition, timely recognition and management of patients with postoperative complications might be influenced or hampered by low staffing levels. Previous research has highlighted the importance of investigating context and safety climate to enable health care systems around the world to improve quality of care, especially in the surgical context.

### Conceptual framework

According to Schneider [21], safety climate can be defined as shared perceptions about the importance of safety to the organization, which are communicated through the attitudes and behaviors that are expected, supported, and rewarded in the work environment. An optimal safety climate is characterized by the following five attributes:

1. The work environment must facilitate safety compliance by providing conditions that maximize consistent adherence to safety related policies and procedures [22].
2. The work environment must be conducive to employee participation in safety. The difference between safety participation and safety compliance lies in its emphasis on voluntary as opposed to required behaviors through which employees contribute positively to workplace safety [22].
3. The organizational response to errors is critical to an optimal safety climate [23, 24].
4. Managerial commitment to safety is essential for an optimal safety climate [25, 26].
5. The work group plays a role in communicating tacit information about behavioral expectations in the workplace [27–30].

Thus safety climate is made up of different dimensions, e.g., leadership, communication, organizational learning, teamwork and attitudes towards the importance of safety [31]. A poor safety climate, stress and knowledge deficits are factors that might negatively affect patient safety [32]. However, knowledge deficits can be prevented by translating evidence-based knowledge into practice and by adopting an evidence-based approach, where leadership and workplace culture are essential factors in knowledge translation and utilization. Successful implementation of evidence-based practice (EBP) depends on evidence, context and facilitation. Context refers to the environment and comprises culture, leadership and evaluation [33], which are also essential aspects of patient safety [31, 32].

EBP for nurses involves carrying out assessments and making decisions based on research, theory, clinical experience and patients’ preferences [34]. EBP is especially important in surgical care because it is considered a high risk area for adverse events [35]. Of the utmost importance in current Swedish health care is to adopt a person centered approach toward patients. Person centeredness is viewed as a core competence in nurses and a prerequisite for patient participation where the patients’ preferences are clearly specified [36]. We argue that person centered care (PCC) is a key component in both patient safety and EBP as it takes the patient perspective into account when making decisions about nursing care. In this study, PCC is considered to be measured by the Context Assessment Index (CAI) factor of “respect for the person”. To our knowledge, there is no research in the surgical area that explored and compared the safety climate and readiness to translate knowledge into practice among nurses. Hence, the aim of this study was to explore the safety climate and readiness to implement evidence and PCC as perceived by registered nurses in Swedish surgical care. Six main hypotheses were tested:

- there is a positive relationship between the safety climate and readiness for EBP
- older surgical nurses report a better attitude toward safety than their younger colleagues
- longer professional experience is associated with better attitudes towards safety
- a higher academic degree is associated with better attitudes towards safety and readiness to implement evidence into practice
- more PCC leads to a better attitude toward safety
- experience and attitudes toward safety and evidence on the part of leadership influence the safety attitude of staff members

## Methods

The design was an exploratory, cross-sectional survey carried out on a national basis. Data were collected from April 2013 to February 2014. The study was conducted in surgical in-patient units with a range of general adult surgical diagnoses and disciplines in all university hospitals in Sweden. Orthopedic and transplantation wards were excluded owing to their unique perspectives on safe care and high risk surgery, while wards dealing with soft tissue surgery were included. The inclusion criterion for questionnaire distribution was RNs active in clinical care in these wards. RNs working full time in administration and/or organizational leadership, such as matrons and head nurses, were excluded. RNs on sick leave, parental leave or leave of absence for other reasons during the data collection period were also excluded.

Permission to distribute the survey to the RNs’ personal mailbox on the ward was obtained from the head nurse at the units. A postage prepaid envelope in which to return the survey was attached. Consent to participate in the study consisted of returning the questionnaire. Two reminders in the form of an e-mail to the head nurse at each unit were sent after 2 and 4 weeks.

**Fig. 1:**
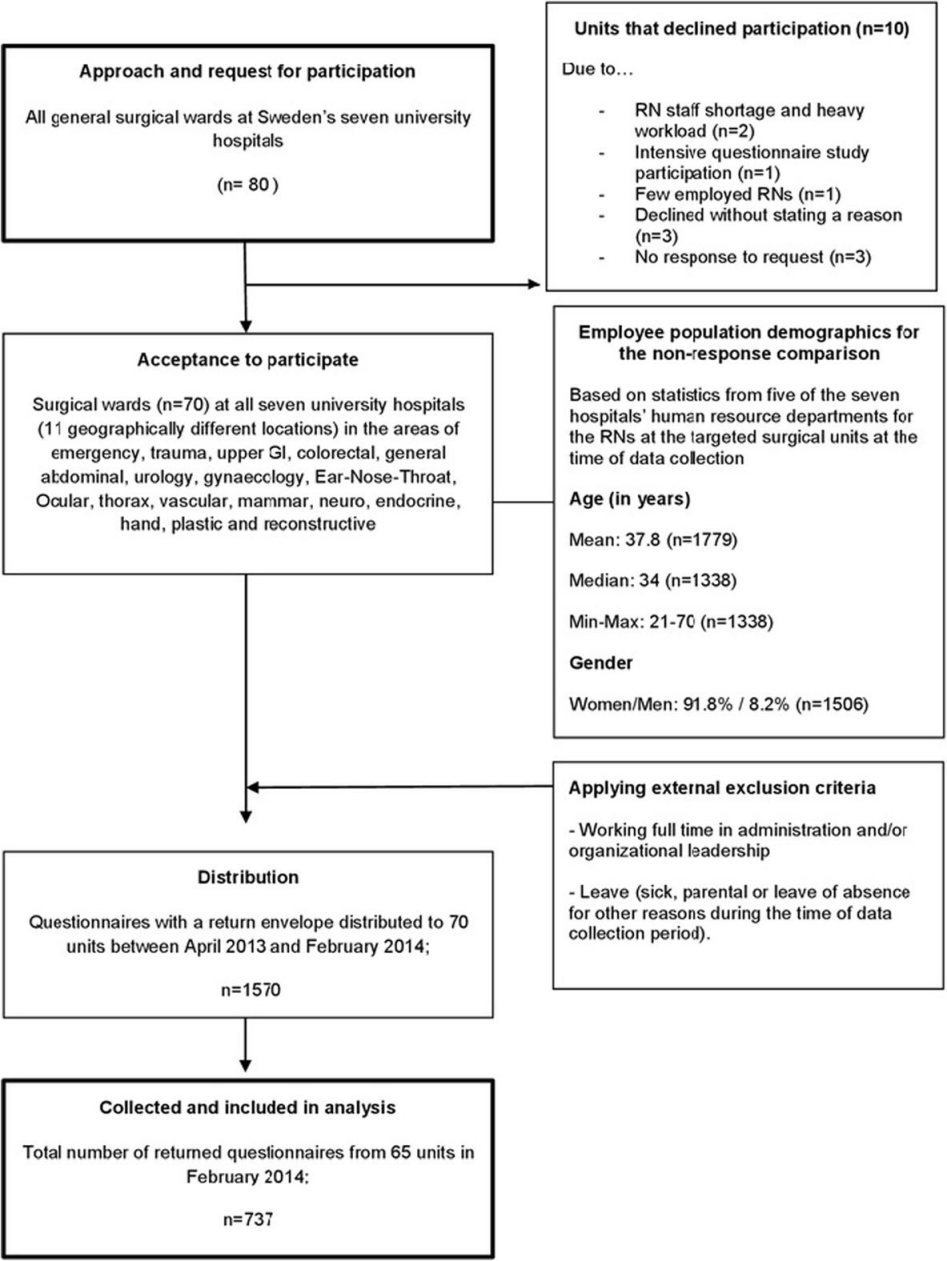
Data collection flow chart

All units (*n* = 80) at the seven university hospitals were contacted and RNs at 70 surgical units were recruited (Fig. 1). In total, 1570 questionnaires were distributed, of which 727 were returned (response rate of 46.3 %). The Human Resource (HR) department at each university hospital was contacted with a request for demographic data concerning the age and gender of the whole population of RNs in all 80 surgical wards. Of the seven university hospital HR departments, five provided these data.

### Instruments

Data were collected by means of two questionnaires, the Safety Attitudes Questionnaire (SAQ – Short form) and the Context Assessment Index (CAI).

The SAQ was originally developed in the USA, after which it was translated and tested for validity and reliability in the Swedish health care context [9]. This instrument allows staff to self-report perceived levels of the safety climate and maturity of the culture. The generic nature of the SAQ facilitates useful comparisons across health care sectors. According to the review of surveys measuring patient safety climate [37], it is the only instrument that investigates safety attitudes where a relationship is shown between the quality of the safety climate, medical errors and shorter hospital stay. The Swedish translation of the SAQ demonstrated good psychometric properties [9, 38] and can be divided into six SAQ dimensions (teamwork, safety, job satisfaction, stress, management, working conditions) measured by 35 statements answered on a 5-point Likert scale (1 = disagree strongly, 2 = disagree slightly, 3 = neutral, 4 = agree slightly, 5 = agree strongly). A sum of ≥60 % positive answers (“agree slightly” and “agree strongly”) in each of the six SAQ dimensions is perceived as acceptable, and lower levels indicate a need for improvement.

The second instrument used was CAI, which investigates the readiness of a practice context for research utilization and stems from the Promoting Action on Research Implementation in Health Services Framework (PARIHS) [39]. CAI is unique as it includes several items (*n* = 7) concerning patient participation and individualized care as part of the context. In this study these items will be referred to as PCC. Context is described as the environment or setting in which people receive health services. The CAI has been translated into Swedish and psychometrically tested in the Swedish health care context [40]. It comprises 37 statements answered on a 4-point Likert scale (1 = strongly disagree, 2 = disagree, 3 = agree, 4 = strongly agree). It is possible to construct the CAI summary and analysis into five factors related to practice (collaborative, evidence-informed, respect for the person, practice boundaries, evaluation), as well as three elements (culture, leadership, and evaluation). The CAI was initially developed in non-acute elderly care, but the Swedish version was later adapted and adjusted for acute care settings and is perceived as neutral vis- à*-*vis the type of care provided. The Swedish version of the CAI has been shown to be easy to answer, comprising patient centered care components, but has been criticized for the risk of question overlap [40].

In addition to the SAQ and CAI, demographic variables were also collected: gender, age, years as a RN, working percent of full time, shift work, highest level of education, and specific education in evidence-based care.

### Analysis

The returned questionnaires were entered and compiled in both Microsoft Excel® (version 2013) and IBM SPSS (Version 21.0). Data are presented as Means, Standard Deviation (SD), Median and Range (minimum and maximum), and in percentages (%). However, a more detailed description of the data presentation of the instruments is required.

Concerning question values, in both the SAQ and the CAI each response alternative for every has a number of magnitude; for the SAQ: 1 = disagree strongly, 2 = disagree slightly, 3 = neutral, 4 = agree slightly, 5 = agree strongly; for the CAI: 1 = strongly disagree, 2 = disagree, 3 = agree, 4 = strongly agree. These values were used as true values. Within each of the dimensions, factors, elements, as well as for the total score, the mean value was calculated and presented in the result section in its interval (SAQ: 1.00 to 5.00, CAI: 1.00 to 4.00). The mean of the true values for each participant within the dimensions, factors, elements, and total score was also used for the statistical analysis (see below).

In accordance with previous research, the percentage of positive answers in the dimensions, factors, elements, and total score was calculated as follows; Firstly, positive answers to each question in both the SAQ and the CAI were assigned a value of 1 and non-positive answers a 0. Positive answers in the SAQ comprised alternatives four (“slightly agree”) and five (“fully agree”), and in the CAI alternatives three (“agree”) and four (“fully agree”) in the 4-step Likert scale (1 = “totally disagree” to 4 = “fully agree”) after adjustment for positive attitudes. Secondly, all answers within the dimensions, factors, elements, and total score were summarized and then divided by the number of questions, resulting in a percentage of positive answers. The percentage of positive answers is presented with the mean true values in a comparative overview of the results.

Statistical analysis was performed in IBM SPSS (Version 21.0). Due to the distribution of the data as well as their nonparametric nature, only nonparametric tests were used. The Spearman rank correlation coefficient (r_s_) was applied to investigate the correlation of demographic factors such as age, years as a RN, and working percent of full time, with the true total SAQ and CAI score, the six SAQ dimensions, the five CAI factors and the three CAI elements, as well as for correlations between the SAQ and the CAI. A weak correlation coefficient (r_s_) interval was set between 0.40 and 0.60 and a strong r_s_-interval over ≥0.61. The Mann-Whitney *U* test was used to investigate differences between RNs with and without a Bachelor degree based on the mean true scores of the SAQ and CAI. The Kruskal–Wallis one-way analysis of variance was used to compare the mean true score of the SAQ and CAI among RNs on different work shifts. Due to the risk of multi-significance, the *p*-value was set at ≤0.01.

### Ethics

Approval for the study was granted by the Regional Ethical Review Board in Gothenburg, Sweden (Dnr.1010-12).

## Results

In total, 1570 questionnaires were distributed to 70 surgical units at Sweden’s seven university hospitals, and 727 were returned (response rate 46.3 %) from 65 units. The participants’ mean age was 36.8 years with a mean time as an RN of 9.7 years. Only 8.4 % of the participants were men. Approximately 66.8 % of the participants had a Bachelor degree or higher, but only 18.5 % had some type of education in evidence-based care. Demographics are presented in Table 1.

**Table 1:** Demographics of the study participants

Gender (man/woman), *n* = 726		8.4 %/91.5 %
Age In years (Mean (SD); Median (Min-Max)), *n* = 718		36.8 (11.2); 34 (22–65)
Years as an RN (Mean (SD); Median (Min-Max)), *n* = 722		9.7 (9.9); 6 (0–46)
Work rate (Mean (SD); Median (Min-Max)), *n* = 722		93.4 %(12.1);100 %(6–100 %)
Work shifts, *n* = 725	Day shift (working hours anywhere between 06:30 AM and 10:00 PM)	53.5 %
	Night shift (working hours anywhere between 08:00 PM and 08:00 AM	12.6 %
	Combined shifts (working hours any time during the day)	33.8 %
Highest level of education, *n* = 723	Professional degree as a Registered Nurse	33.2 %
	Bachelor in nursing science	57.3 %
	Magister (European Master first year) in nursing science	7.1 %
	Master (Full European Master 2 years) in nursing science	0.1 %
	Doctoral degree (PhD)	0.1 %
	Other education at advanced or doctoral level	2.2 %
Specific education in Evidence based care (EBC), *n* = 714	Unknown	8.7 %
	No education in EBC	72.8 %
	Yes, short course or work based education	8.8 %
	Yes, university course	9.7 %

When comparing the population data retrieved on RNs employed at five of the seven university hospitals’ HR units (as presented in Fig. 1), the mean and range of age and gender distribution did not differ significantly from the demographics of the study population. Hence, the participants can be considered representative of the target population in terms of age and gender distribution. However, due to the nature of the data received from the HR departments, statistical comparison between the study sample and the total RN population was not possible.

Concerning the overview of the SAQ, the responses were calculated and stratified into a total score and the six SAQ dimensions. In the overview of the CAI, responses were calculated and stratified into a total score, as well as the five CAI factors described by McCormack et al. in “Development and Testing of the Context Assessment Index (CAI)” [39] and the three CAI elements described by McCormack in the “Guide to using the context assessment index (CAI)” from the University of Ulster/University College Cork [41]. True score and positive ratings both for the SAQ total score and dimensions, as well as the total score, factors and elements in the CAI are presented in Table 2.

**Table 2:** Summary of the mean true score and the positive rating of the current safety climate (SAQ) and readiness for evidence-based practice and person centered care (CAI). The total SAQ score, the six SAQ dimensions, the total CAI score, the five CAI factors and three CAI elements are presented

			Mean true score	Percentage of participants who gave positive answers about their current situation
			Mean (SD)	Mean (SD)
			Median (min-max)	Median (min-max)
		Total SAQ rating (*n* = 502)	3.9 (0.5)	71.3 % (18.4 %)
			4 (1–5)	74.3 % (0–100 %)
SAQ	Six SAQ dimensions	Teamwork climate (*n* = 694)	4.1 (0.6)	80.6 % (22.2 %)
			4 (1–5)	83.3 % (0–100 %)
		Safety climate (*n* = 624)	3.8 (0.7)	65.7 % (27.0 %)
			4 (1–5)	71.4 % (0–100 %)
		Job satisfaction (*n* = 710)	4.2 (0.7)	82.7 % (26.2 %)
			4 (1–5)	100 % (0–100 %)
		Stress recognition (*n* = 689)	4.0 (0.9)	76.9 % (29.7 %)
			4 (1–5)	100 % (0–100 %)
		Perceptions of management (*n* = 623)	3.6 (0.9)	58.3 % (35.3 %)
			4 (1–5)	60 % (0–100 %)
		Working conditions (*n* = 674)	3.5 (0.8)	60.9 % (32.9 %)
			4 (1–5)	75.0 % (0–100 %)
CAI		Total CAI rating (*n* = 639)	2.9 (0.4)	73.4 % (19.6 %)
			2 (1–4)	75.5 % (13.9–100 %)
	Five CAI factors	Collaborative practice (*n* = 704)	2.9 (0.4)	72.2 % (24.8 %)
			2 (1–4)	77.8 % (0–100 %)
		Evidence-informed practice (*n* = 666)	2.8 (0.5)	66.9 % (26.4 %)
			2 (1–4)	72.7 % (0–100 %)
		Respect for the person (*n* = 709)	3.2 (0.4)	88.6 % (16.5 %)
			2 (1–4)	100 % (0–100 %)
		Practice boundaries (*n* = 707)	3.0 (0.5)	78.1 % (23.9 %)
			2 (1–4)	83.3 % (0–100 %)
		Evaluation (*n* = 712)	2.7 (0.6)	59.8 % (29.6 %)
			2 (1–4)	50.0 % (0–100 %)
	Three CAI elements	Culture (*n* = 679)	2.9 (0.4)	70.4 % (21.1 %)
			2 (1–4)	75.0 % (0–100 %)
		Leadership (*n* = 699)	2.9 (0.4)	76.1 % (22.3 %)
			2 (1–4)	87.5 % (0–100 %)
		Evaluation (*n* = 678)	2.9 (0.4)	75.5 % (21.5 %)
			2 (1–4)	78.6 % (7.1–100 %)

Notable in the rating of the six SAQ dimensions is that “perception of management”, i.e., approval of managerial action, indicated a need for improvement, and “safety climate”, i.e., perception of a strong and proactive organizational commitment to safety, and “working conditions”, i.e., perceived quality of the work environment and logistical support, indicated a threshold level for a non-acceptable climate. The CAI does not have any stated margin for “good” or “bad”. However, it is notable that in the CAI factor summary “evaluation” was perceived as the weakest of the five factors, followed by “evidence informed practice”.

### Evidence-based practice, person centered care, and safety climate

Before this study we hypothesized that there is a positive relationship between safety climate and readiness for implementation, i.e., EBP and PCC. We assumed that more EBP and PCC would lead to a better attitude toward safety. We also believed that experienced leadership and its attitudes toward safety and evidence would influence the surgical RNs’ own attitude to safety. The analysis revealed a strong correlation between the mean true total SAQ score and the mean true total CAI score (r_s_ = 0.70; *p* < 0.0001; *n* = 454). Correlations between the six SAQ dimensions, the five CAI factors, and the three CAI elements are presented in Table 3. Overall, a weak to strong correlation was found between the instruments’ strata, with the exception of “stress recognition” in the SAQ, which indicates r_s_-values that have no correlation with the five factors and the three elements of the CAI. The most pronounced relationships were found between perception of management (SAQ) and evidence informed practice (CAI) (r_s_ = 0.66; *p* < 0.001; *n* = 577), culture (CAI) (r_s_ = 0.66; *p* < 0.001; *n* = 587), evaluation (CAI) (r_s_ = 0.63; *p* < 0.001; *n* = 586), and practice boundaries (CAI) (r_s_ = 0.61; *p* < 0.001; *n* = 610), between safety climate (SAQ) and culture (CAI) (r_s_ = 0.63; *p* < 0.001:*n* = 589), evidence informed practice (CAI) r_s_ = 0.61; *p* < 0.001: *n* = 577) and evaluation (CAI) (r_s_ = 0.60; *p* < 0.001; *n* = 586), and between teamwork climate (SAQ) and practice boundaries (CAI) (r_s_ = 0.61; *p* < 0.001; *n* = 676) and evaluation (CAI) (r_s_ = 0.61; *p* < 0.001; *n* = 651), suggesting a perception among Swedish surgical nurses that a strong and proactive organizational commitment to safety is strongly related to a culture guided by EBP, where evaluation takes place on a regular basis.

**Table 3:** Correlation between the mean true score of the six SAQ dimensions, the five CAI factors, and the three CAI elements

			SAQ					
			Six SAQ dimensions					
			Teamwork climate	Safety climate	Job satisfaction	Stress recognition	Perceptions of management	Working conditions
CAI	Five CAI factors	Collaborative practice	r_s_ = 0.53	r_s_ =0.52	r_s_ =0.49	r_s_ = -0.18	r_s_ =0.52	r_s_ =0.42
			(*p* < 0.0001)	(*p* < 0.0001)	(*p* < 0.0001)	(*p* < 0.0001)	(*p* < 0.0001)	(*p* < 0.0001)
			*n* = 674	*N* = 605	*n* = 687	*n* = 668	*n* = 604	*n* = 652
		Evidence informed practice	r_s_ =0.57	r_s_ =0.61	r_s_ =0.53	r_s_ = -0.21	r_s_ =0.66	r_s_ =0.55
			(*p* < 0.0001)	(*p* < 0.0001)	(*p* < 0.0001)	(*p* < 0.0001)	(*p* < 0.0001)	(*p* < 0.0001)
			*n* = 640	*n* = 577	*n* = 652	*n* = 635	*n* = 577	*n* = 620
		Respect for the person	r_s_ =0.58	r_s_ =0.51	r_s_ =0.51	r_s_ = -0.16	r_s_ =0.52	r_s_ =0.43
			(*p* < 0.0001)	(*p* < 0.0001)	(*p* < 0.0001)	(*p* < 0.0001)	(*p* < 0.0001)	(*p* < 0.0001)
			*n* = 677	*n* = 608	*n* = 692	*n* = 673	*n* = 607	*n* = 657
		Practice boundaries	r_s_ =0.61	r_s_ =0.58	r_s_ =0.58	r_s_ = -0.17	r_s_ =0.61	r_s_ =0.49
			(*p* < 0.0001)	(*p* < 0.0001)	(*p* < 0.0001)	(*p* < 0.0001)	(*p* < 0.0001)	(*p* < 0.0001)
			*n* = 676	*n* = 610	*n* = 692	*n* = 671	*n* = 610	*n* = 657
		Evaluation	r_s_ =0.44	r_s_ =0.50	r_s_ =0.43	r_s_ = -0.18	r_s_ =0.53	r_s_ =0.46
			(*p* < 0.0001)	(*p* < 0.0001)	(*p* < 0.0001)	(*p* < 0.0001)	(*p* < 0.0001)	(*p* < 0.0001)
			*n* = 682	*n* = 614	*n* = 696	*n* = 675	*n* = 612	*n* = 660
	Three CAI elements	Culture	r_s_ =0.59	r_s_ =0.63	r_s_ =0.57	r_s_ = -0.21	r_s_ =0.66	r_s_ =0.56
			(*p* < 0.0001)	(*p* < 0.0001)	(*p* < 0.0001)	(*p* < 0.0001)	(*p* < 0.0001)	(*p* < 0.0001)
			*n* = 651	*n* = 589	*n* = 663	*n* = 646	*n* = 587	*n* = 630
		Leadership	r_s_ =0.57	r_s_ =0.56	r_s_ =0.51	r_s_ = -0.18	r_s_ =0.56	r_s_ =0.43
			(*p* < 0.0001)	(*p* < 0.0001)	(*p* < 0.0001)	(*p* < 0.0001)	(*p* < 0.0001)	(*p* < 0.0001)
			*n* = 669	*n* = 605	*n* = 683	*n* = 665	*n* = 602	*n* = 648
		Evaluation	r_s_ =0.61	r_s_ =0.60	r_s_ =0.58	r_s_ = -0.22	r_s_ =0.63	r_s_ =0.52
			(*p* < 0.0001)	(*p* < 0.0001)	(*p* < 0.0001)	(*p* < 0.0001)	(*p* < 0.0001)	(*p* < 0.0001)
			*n* = 651	*n* = 586	*n* = 664	*n* = 646	*n* = 586	*n* = 631

### Age, professional experience and assigned working shift

We hypothesized that older surgical nurses and those with longer professional experience would report a better attitude towards safety than their younger colleagues with less professional experience. We further assumed that the safety climate might be perceived differently depending on the shift worked. Hence, we investigated the correlation between age, years as an RN and the percentage of full time work in the total SAQ and CAI scores, as well as each of the six SAQ dimensions, the Five CAI factors and the Three CAI elements (see Additional file 1: Table S4). No significant r_s_-value indicated any correlation of importance, suggesting that these factors do not influence the dimensions, factors or elements when used in isolation.

In a comparison between the three work shifts and the rating of the total SAQ and CAI score, as well as the six SAQ dimensions, five CAI factors and three CAI elements (see Additional file 2: Table S5), it was seen that the night shift nurses rated the total SAQ score significantly lower (*p* = 0.002; *n* = 500) with a 0.3 mean true score, the SAQ safety climate (*p* = 0.003; *n* = 622) lower by a 0.4 mean true score, and the CAI factor of practicing boundaries (*p* = 0.005;*n* = 706) lower by a 0.2 mean true score, compared to nurses on the day shift or combined shift. No significant differences were observed between the day and the combined shift (*p* > 0.50).

Although not statistically significant, there was a trend that all measurements (see Additional file 2: Table S5), with the exception of the CAI element Culture, had lower ratings from night shift nurses in comparison to those working on the day shift and combined shift.

### Academic degree, safety climate and readiness to implement evidence into practice

One initial research question was whether a higher academic degree and education in evidence-based care improve attitudes to safety and readiness to implement evidence into practice. In a comparison of participants who only had a nursing degree and participants with at least a Bachelor’s degree in terms of the total SAQ score and the six SAQ dimensions (see Additional file 3: Table S6), a significant difference was observed in safety climate (*p* < 0.001;*n* = 608), stress recognition (*p* < 0.0001;*n* = 672) and working conditions (*p* < 0.0001;*n* = 655), with lower ratings on safety climate and working conditions, and higher rating in stress recognition from participants with a Bachelor degree in nursing science. In the same CAI analysis (see Additional file 3: Table S6),), there was a significant difference (*p* < 0.0001; *n* = 692) in the factor evaluation, indicating that participants with a Bachelor degree rated evaluation lower than participants without a Bachelor degree in nursing science.

The difference between participants with and without any form of education in evidence-based care was also investigated in relation to the total SAQ score, the six SAQ dimensions, the total CAI score, the five CAI factors and the three CAI elements. No statistical difference (*p* > 0.08) or distributional difference was observed in the data.

Finally, we analyzed the perception of stress recognition in the work place measured as a dimension of the SAQ (see Additional file 1: Table S4). Correlating the analysis to age and years as an RN indicated no statistically significant relationship (age: r_s_ = -0.16; *p* < 0.001; *n* = 680) (years as RN: r_s_ = -0.16; *p* < 0.001; *n* = 684).

## Discussion

This is the first Swedish nationwide survey aimed at exploring the safety climate in surgical units as perceived by RNs and the study is thus unique. University hospitals are recognized for their high standards in research, education, and specialized care. In general, we expect research utilization in university hospitals to be good and the culture to be characterized by EBP. It is possible to argue that readiness for EBP is a psycho-social measure affected by, among other things, age and experience [42–44], that has little relevance to what happens when someone introduces a new process into the hospital. However, our findings reflect the culture within a high risk area of hospital care, thus the location and context make them highly relevant.

The first hypothesis regarding a presumed positive relationship between safety climate and readiness for implementation, i.e., EBP and PCC, was confirmed. However, it is worth noting that the findings presented in Table 3 reveal relationship patterns between EBP and PCC to each of the columns that basically explain all of the variance of the SAQ dimensions. However, patient safety depends on a favorable context for RNs [45, 46] as efforts to increase the quality of the work context are a way of reducing the frequency of hospital acquired infections. It has previously been shown that the possibility of the patient dying within 30 days of admission is 14 % lower in hospitals where RNs rate their work context higher than in hospitals with lower ratings [47, 48]. In our study, job satisfaction and teamwork climate were rated >80 %, suggesting a favorable context for surgical nurses despite the fact that the perception of management indicated a need for improvement. “Safety climate”, i.e., the perception of a strong and proactive organizational commitment to safety, and “working conditions”, i.e., perceived quality of the work environment and logistical support, also had low ratings, although still over the 60 % level required for an acceptable safety climate.

The hypothesis that older surgical nurses possess a better attitude towards safety than their younger colleagues was not confirmed. Neither age nor work experience seemed to affect safety attitudes. This in contrast to previous research by Raftopoulos and Pavlakis [49] on Greek-Cypriot intensive care nurses whereas SAQ score positively correlated to age, indicating that older nurses rated their safety attitude higher compared to their younger collages. Also, concerning the use of EBP, Dahlheim et al. [42] report that older Norwegian nurses rated applying EBP more extensively then there younger collages. However, our findings might be explained by findings from the study by Förberg et al. [50], which described RNs’ adherence to a clinical practice guideline for peripheral venous catheters. An unexpected finding in their study was that the use of disposable gloves among RNs with long work experience was related to lower adherence. This suggests that extensive work experience might lead to a more relaxed attitude to patient related safety interventions in clinical practice, which is the opposite to our assumption and previous research findings that more experienced nurses are more careful about protecting the patient from harm.

We hypothesized that the safety climate might be perceived differently depending on the shift worked. This assumption was confirmed, as nurses on the night shift rated the SAQ total score and safety climate as well as the CAI factor of practicing boundaries significantly lower in comparison to those on the day shift or combined shift. This indicates that the nurses working the night shift would not feel safe being a patient in their own unit but also that practice boundaries were perceived as less problematic on the night shift. An overall non-statistically significant trend in terms of lower ratings was observed in nurses who worked on the night shift. It is known that night shift nurses have a greater need of teamwork and reliance on each other due to low staffing levels [51]. This is especially relevant in the surgical unit, as the surgeon is often absent or occupied in the theatre or the emergency department. It has been suggested that nurses who work the night shift would benefit from more support and education regarding the safety aspects of care [52, 53]. To our surprise, current research literature states combined shift work affects nurses more strongly than working exclusively on the day or night shift [54]. However, there was no indication of lower ratings of the SAQ or CAI properties among the participants who worked shifts in the present study.

We analyzed whether more PCC is related to a better attitude to safety. Respect for the person (in this study viewed as PCC) was moderately related to teamwork climate, safety climate, perception of management, job satisfaction and working conditions. Sexton et al. [14] also highlighted the importance of organizational cultural attitudes toward teamwork behavior but did not discuss whether good teamwork promotes PCC. One of our hypothesis was that more PCC leads to a better attitude to safety. Possible risks can be identified by inviting the patient to participate and listening to her/his narrative, thus ensuring better patient safety. Considering the patient as part of the health care team is vital for the safety climate in general and for EBP in particular [34]. Establishing PCC relationships most likely improves job satisfaction in the surgical context with its high patient turn over. According to a review by Suhonen et al. [55], matters associated to the ethics of an individual nurse and the work environment provided by the organization constitute the main driving force for individualized care. The link between the work environment and safety climate has been clearly established in the literature [18].

One assumption was that university hospitals employ nurses with a high academic degree and that a higher academic degree improves attitudes to safety and readiness for implementation of evidence into practice. In the SAQ analysis, RNs with a Bachelor degree gave a lower rating to the safety climate and working conditions, but rated stress recognition higher than RNs without a bachelor degree. Nurses with a Bachelor degree also rated evaluation lower than did nurses with only a degree in nursing. In addition, a trend toward lower ratings of the CAI element of culture was found among RNs with a Bachelor degree or higher compared to RNs with only a nursing degree. According to PARIHS [33], the more favorable the context, the better the conditions for implementation. High scores mean that the surgical nurses consider the context receptive to change, as it represents a more sympathetic culture, stronger leadership and useful evaluative systems [33]. Aiken et al. [56] clearly demonstrated that staffing by nurses with a Bachelor degree is a highly protective factor against patient mortality after surgery. Combining this results to our findings, this might be explained by the fact that nurses with a high academic degree adopt a critical approach towards their practice and context. As a consequence, they demand an adequate safety climate and good working conditions, as well as probably being more dissatisfied with poor evaluation as it hinders EBP. It is very important to focus on optimizing care to prevent surgery related complications. However, it has also been highlighted that when complications arise, the care must be optimally managed and evidence based [20]. Nurse managers play a vital role in promoting EBP and good working conditions. Several Swedish studies have shown that a lack of academic education, research use, and the failure of nurse managers to support such use are associated with less EBP [43, 57, 58]. Nurses with a high academic degree were also less satisfied with their working conditions, which indicated a need for improvement in the overall results.

Finally, we assumed that experienced leadership and its attitudes toward safety and evidence might affect the safety attitude of individual RNs. The relationship between the dimensions of leadership and safety climate was strong (r_s_ = 0.70; *p* < 0.0001; *n* = 454). Leadership at all levels of an organization has been found to influence RNs’ use of clinical practice guidelines, which in general are developed to promote safe care [59]. Sredl et al. [60] studied nurse managers’ belief in EBP and its implementation in hospitals in the USA. The results revealed that although the majority of nurse managers had strong belief in the value of EBP, the actual implementation was relatively low. In their conclusion, Sredl et al. [60] stated that nurse managers must create a culture of acceptance for EBP, and act as agents of change. Based on our findings we argue that their conclusion is definitely transferrable to the area of safety climate in Swedish surgical care.

## Limitations

This study focused on nurses in surgical care wards in Sweden’s seven university hospitals. Paper questionnaires were used for the assessment and a total response rate of just over 46 % was achieved. We consider this to be acceptable in a Swedish study, although it might have been higher had a mixed mode been used, i.e., allowing the participants to choose between a paper and a web based questionnaire, based on the findings of Greenlaw and Brown-Welty [61].

Two reminders were sent by e-mail to the head nurses or matrons. It can be assumed that additional reminders may have helped to increase the response rate [62]. Reminders sent directly to the target population via e-mail would improve the response rate even more. As indications of decreasing response rates have been emphasized in the literature [63], a 46 % response rate can be considered acceptable. However, the results may be interpreted as being based on under half of the population as opposed to a controlled sample. A strength of the data is that of the total number of wards (*n* = 80), 81 % (*n* = 65) participated, meaning that the results cover more than three fourths of the whole target population. When compared with the demographic data on the age and gender of the total nursing population received from the HR units of five of the seven university hospitals, the distribution seems to be similar. However, the fact that data from two university hospitals are missing and statistical comparative calculations were not possible could also constitute a limitation.

There are undoubtedly other factors, e.g., how busy the hospital was, how ill the patients were, the management history, and productivity pressure, which were not measured in this study but may explain some of the findings. We recommend further studies with additional measurements and analysis of the four factors mentioned above, which was not possible in this study due to the Swedish state funded health care system that lacks internal and external competitive elements and the fact that the ethical approval only included results presentation on demographical group levels.

## Conclusions

In conclusion, this study presents new knowledge regarding the safety climate and readiness to implement EBP and PCC in general surgical wards in university hospitals and indicates important associations between these two areas. The perceived safety climate is positively correlated with EBP and PCC. While RNs generally reported positive job satisfaction and a good team work culture in their units, there were indications that improvements in organizational management are needed.

## Additional files

Additional file 1: Table S4. Correlation between age, years as an RN and current percent of full time work in the mean true score of the SAQ total score, each of the six SAQ dimensions, total CAI score, the five CAI factors and the three CAI elements. (DOCX 14 kb) Additional file 2: Table S5. Summary of mean values and standard deviation for the mean true score between work shifts, presented by the total SAQ score, the six SAQ dimensions, the total CAI score, the five CAI factors, and three CAI elements. (DOCX 14 kb) Additional file 3: Table S6. Summary of mean values and standard deviation for the mean true score between participants with and without a bachelor degree (BSc) in nursing science, presented by the total SAQ score, the six SAQ dimensions, the total CAI score, the five CAI factors, and three CAI elements. (DOCX 14 kb)

## Abbreviations

CAI: The Context Assessment Index
CO: Camilla olsson
EBP: Evidence based practice
NSQIP: The National Surgical Quality Improvement Program
PARISH: The Promoting Action on Research Implementation in Health Services Framework
PCC: Person centered care
RN: Registered nurse (Swe: Legitimerad Sjuksköterska)
r_s_: Spearman rank correlation coefficient
SAQ: The Safety Attitudes Questionnaire
SD: Standard deviation.
