# Supplementary material for: Safety climate and readiness for implementation of evidence and person centered practice – A national study of registered nurses in general surgical care at Swedish university hospitals
Source: BMC Nurs. 2016 Sep 13;15(1):54. doi: 10.1186/s12912-016-0174-2 (PMC5020433; doi:10.1186/s12912-016-0174-2)
Supplement: Additional file 1: Table S4. — Correlation between age, years as an RN and current percent of full time work in the mean true score of the SAQ total score, each of the six SAQ dimensions, total CAI score, the five CAI factors and the three CAI elements. (DOCX 14 kb) [file 12912_2016_174_MOESM1_ESM.docx]

**Table 4**. Correlation between age, years as an RN and current percent of full time work in the mean true score of the SAQ total score, each of the six SAQ dimensions, total CAI score, the five CAI factors and the three CAI elements.

|  | | | Age in years | Years as RN | Percent of full time work |
| --- | --- | --- | --- | --- | --- |
| SAQ |  | Total SAQ rating | r_s_=0.10  (p=0.018)  n=496 | r_s_=0.08  (p=0.064)  n=497 | r_s_ =0.03  (p=0.556)  n=498 |
|  | Six SAQ dimensions | Teamwork climate | r_s_ =0.04  (p=0.918)  n=686 | r_s_ =0.02  (p=0.632)  n=689 | r_s_ <0.01  (p=0.816)  n=690 |
|  |  | Safety climate | r_s_ =0.17  (p=0.001)  n=616 | r_s_ =0.12  (p=0.002)  n=619 | r_s_ =0.01  (p=0.784)  n=620 |
|  |  | Job satisfaction | r_s_ =0.03  (p=0.375)  n=701 | r_s_=-0.01  (p=0.875)  n=705 | r_s_ =0.05  (p=0.201)  n=705 |
|  |  | Stress recognition | r_s_ =-0.16  (p<0.0001)  n=680 | r_s_ =-0.16  (p<0.0001)  n=684 | r_s_ =0.06  (p=0.123)  n=684 |
|  |  | Perceptions of management | r_s_ =0.12  (p=0.003)  n=615 | r_s_ =0.10  (p=0.017)  n=618 | r_s_ =0.03  (p=0.426)  n=618 |
|  |  | Working conditions | r_s_ =0.16  (p<0.0001)  n=666 | r_s_ =0.13  (p=0.001)  n=669 | r_s_ =-0.05  (p=0.225)  n=670 |
|  | | Total CAI rating | r_s_ <0.01  (p=0.911)  n=636 | r_s_ =-0.04  (p=0.294)  n=637 | r_s_ =-0.01  (p=0.873)  n=637 |
| CAI | Five CAI factors | Collaborative practice | r_s_ =-0.04  (p=0.346)  n=696 | r_s_ =-0.06  (p=0.097)  n=700 | r_s_ =0.01  (p=0.774)  n=700 |
|  |  | Evidence informed practice | r_s_ = -0.01  (p=0.796)  n=661 | r_s_ =-0.04  (p=0.302)  n=663 | r_s_ <0.01  (p=0.947)  n=663 |
|  |  | Respect for the person | r_s_ =-0.04  (p=0.250)  n=701 | r_s_ =-0.06  (p=0.105)  n=705 | r_s_ =0.02  (p=0.695)  n=705 |
|  |  | Practice boundaries | r_s_ =-0.07  (p=0.053)  n=700 | r_s_ =-0.09  (p=0.017)  n=703 | r_s_ = 0.04  (p=0.247)  n=704 |
|  |  | Evaluation | r_s_ =0.16  (p<0.0001)  n=706 | r_s_ =0.14  (p<0.0001)  n=708 | r_s_ =-0.04  (p=0.272)  n=708 |
|  | Three CAI elements | Culture | r_s_ =0.03  (p=0.423)  n=674 | r_s_ <0.01  (p=0.933)  n=676 | r_s_ =0.01  (p=0.849)  n=677 |
|  |  | Leadership | r_s_ =0.02  (p=0.565)  n=692 | r_s_ =-0.06  (p=0.140)  n=695 | r_s_ <0.01  (p=0.927)  n=695 |
|  |  | Evaluation | r_s_ =-0.03  (p=0.379)  n=673 | r_s_ =-0.07  (p=0.082)  n=675 | r_s_ =0.01  (p=0.824)  n=674 |
