# Supplementary material for: Safety climate and readiness for implementation of evidence and person centered practice – A national study of registered nurses in general surgical care at Swedish university hospitals
Source: BMC Nurs. 2016 Sep 13;15(1):54. doi: 10.1186/s12912-016-0174-2 (PMC5020433; doi:10.1186/s12912-016-0174-2)
Supplement: Additional file 3: Table S6. — Summary of mean values and standard deviation for the mean true score between participants with and without a bachelor degree (BSc) in nursing science, presented by the total SAQ score, the six SAQ dimensions, the total CAI score, the five CAI factors, and three CAI elements. (DOCX 14 kb) [file 12912_2016_174_MOESM3_ESM.docx]

**Table 6.** Summary of mean values and standard deviation for the mean true score between participants with and without a bachelor degree (BSc) in nursing science, presented by the total SAQ score, the six SAQ dimensions, the total CAI score, the five CAI factors, and three CAI elements.

|  | | | RN without BSc  (n=142) | RN with BSc  (n=302) | Mann-Whitney U test |
| --- | --- | --- | --- | --- | --- |
|  |  |  | Mean true score  (Mean (SD)) | Mean true score  (Mean (SD)) |  |
|  | | Total SAQ rating | 3.9 (0.5) | 3.9 (0.5) | p=0.281  (n=489) |
| SAQ | Six SAQ dimensions | Teamwork climate | 4.2 (0.6) | 4.1 (0.6) | p=0.279  (n=676) |
|  |  | Safety climate | 4.0 (0.7) | 3.7 (0.7) | **p<0.001**  **(n=608)** |
|  |  | Job satisfaction | 4.2 (0.8) | 4.2 (0.7) | p=0.198  (n=690) |
|  |  | Stress recognition | 3.8 (0.9) | 4.1 (0.9) | **p<0.001**  **(n=672)** |
|  |  | Perceptions of management | 3.6 (0.9) | 3.5 (0.9) | p=0.210  (n=605) |
|  |  | Working conditions | 3.7 (0.9) | 3.4 (0.8) | **p<0.001**  **(n=655)** |
| CAI |  | Total CAI rating | 3.0 (0.4) | 2.9 (0.4) | p=0.070  (n=623) |
|  | Five CAI factors | Collaborative practice | 2.9 (0.5) | 2.9 (0.4) | p=0.036  (n=686) |
|  |  | Evidence-informed practice | 2.8 (0.5) | 2.8 (0.5) | p=0.160  (n=650) |
|  |  | Respect for the person | 3.2 (0.4) | 3.2 (0.4) | p=0.797  (n=692) |
|  |  | Practice boundaries | 3.0 (0.4) | 3.0 ( 0.5) | p=0.599  (n=690) |
|  |  | Evaluation | 2.9 (0.5) | 2.7 (0.6) | **p<0.001**  **(n=692)** |
|  | Three CAI elements | Culture | 2.9 ( 0.4) | 2.9 (0.4) | p=0.016  (n=662) |
|  |  | Leadership | 3.0 (0.4) | 2.9 (0.4) | p=0.118  (n=682) |
|  |  | Evaluation | 3.0 (0.4) | 2.9 (0.4) | p=0.297  (n=661) |
